# p53 status correlates with the risk of progression in stage T1 bladder cancer: a meta-analysis

**DOI:** 10.1186/s12957-016-0890-9

**Published:** 2016-04-30

**Authors:** Jun Du, Shu-hua Wang, Qing Yang, Qian-qian Chen, Xin Yao

**Affiliations:** Department of Genitourinary Oncology, Tianjin Medical University Cancer Institute and Hospital, National Clinical Research Center for Cancer, Key Laboratory of Cancer Prevention and Therapy, Tianjin, People’s Republic of China

**Keywords:** p53, Non-muscle invasive bladder cancer, Stage T1, Progression, Meta-analysis

## Abstract

**Background:**

Published studies have yielded inconsistent results on the relationship between p53 status and the progression of stage T1 non-muscle invasive bladder cancer (NMIBC). Therefore, we performed a meta-analysis to evaluate the prognostic value of p53 in T1 NMIBC.

**Methods:**

We systematically searched for relevant literatures in MEDLINE, EMBASE, and Web of Science. Data were pooled together from individual studies, and meta-analysis was performed. Study quality was assessed using the Newcastle-Ottawa Scale. Pooled risk ratios (RRs) and 95 % CI were calculated to estimate the effect sizes. Moreover, subgroup analyses were carried out.

**Results:**

A total of 12 studies comprising 712 patients were subjected to the final analysis. p53 overexpression was significantly associated with higher progression rate of T1 NMIBC (RR 2.32, 95 % CI 1.59–3.38). Moderate heterogeneity was observed across the studies (*I*^2^ = 39 %, *P* < 0.0001). In a subgroup analysis stratified by stage, p53 overexpression was a predictor of progression in T1 grade 3 NMIBC (RR 2.71, 95 % CI 1.31–5.64). In addition, in a subgroup analysis stratified by intravesical therapy, p53 overexpression was a predictor of progression in T1 NMIBC received Bacillus Calmette-Guérin intravesical therapy (RR 3.35, 95 % CI 1.89–5.93). Furthermore, after excluding the study that possibly contributed to the heterogeneity by the sensitivity analysis, the association p53 overexpression was significantly correlated with progression of T1 NMIBC (RR 2.74, 95 % CI 2.05–3.65) without evidence of heterogeneity (*I*^2^ = 0 %, *P* < 0.0001).

**Conclusions:**

This meta-analysis suggested that p53 overexpression may be associated with progression of T1 NMIBC patients. Because of the heterogeneity and other limitations, further studies with rigid criteria and large populations are still warranted to confirm our findings.

## Background

There are 386,000 new cases of bladder cancer worldwide every year and caused 150,000 cancer-specific deaths [1]. Approximately 70 % of bladder cancer are non-muscle invasive bladder cancers (NMIBCs) at the time of presentation [2], and stage T1 disease showing invasion of the lamina propria present 25 % of NMIBC [3]. T1 NMIBC represent a clinical challenge because they are inherently aggressive and have heterogeneous outcomes. Up to 50 % of T1 NMIBC managed with intravesical therapy progress to muscle invasive BC (MIBC) within 5 years [4]. Progression rather than recurrence has been associated with increased chance of metastasis and death from systemic disease. The European Organisation for Research and Treatment of Cancer (EORTC) has proposed a scoring system for predicting progression of NMIBC using a weighted variable system including grade (WHO 1973), stage, CIS, multiplicity, size, and previous recurrence rate [5]. Although the EORTC risk score represents a major improvement, it does not fully capture tumor heterogeneity of T1 NMIBC.

Another approach has been to identify biomarkers to predict the probability of progression in T1 NMIBC [6]. Cell cycle modulators are often deregulated in bladder cancer, including alterations in various proteins such as p53, CCNB1, p16, and p27 [6, 7]. p53 is frequently mutated in patients with bladder cancer [8, 9]. Compared with the wild-type protein, mutant p53 proteins have a prolonged half-life and are thus more likely to be detected by immunohistochemical assays [10]. In bladder cancer, because of the high concordance between p53 nuclear immunoreactivity and genomic mutations, immunohistochemistry is a useful surrogate for examining p53 mutation status [9, 11]. The value of pretreatment p53 status on the progression of T1 NMIBC has been studied and discussed. As a result, several studies reported that p53 overexpression is positively associated with progression of T1 NMIBC [12, 13]. However, some studies failed to confirm the association between p53 overexpression and progression of T1 NMIBC [14, 15].

Considering the inconsistent results of published articles, we conducted a meta-analysis to determine the p53 status in predicting progression of T1 NMIBC.

## Methods

### Search strategy

We conducted and reported this meta-analysis following the PRISMA statement. A MEDLINE, EMBASE, and Web of Science search for studies investigating the progression significance of p53 in T1 bladder cancer was performed using the following keywords: [urinary bladder neoplasms] OR [urinary AND bladder AND neoplasms] OR [bladder AND cancer] OR [bladder cancer] AND [T1] AND [p53] OR [TP53] AND [progression]. The final search was conducted on October 10, 2015. Searches were limited to studies published in English. The eligible publications were selected by two reviewers.

### Inclusion and exclusion criteria

Studies were considered eligible if they met the following inclusion criteria: (1) the study included proven diagnosis of urothelial carcinoma; (2) the study considered TURBT as a treatment modality; (3) the study assessed the association between p53 and progression of patients with T1 bladder cancer; (4) to detect p53 status in the primary tumor tissues using immunohistochemistry (IHC); and (5) the study provided the data that showed number of events. Studies were excluded based on any of the following criteria: (1) review articles, commentaries, letters to the editor, or case reports or (2) laboratory studies, such as studies on bladder cancer cell lines and animal models; for overlapping studies, the most recent or most complete study was used to avoid duplication of information.

### Data extraction

According to the inclusion criteria listed above, data were extracted independently by two reviewers for each eligible study. We extracted data including (1) study information including the name of first author, year of publication, sample size, and time of research; (2) patient characters including grade and intravesical therapy; (3) p53 antibody clone and cutoff value of positivity of p53; and (4) progression data (number of events).

### Study quality assessment

Quality assessment of included studies was evaluated by two independent reviewers (DJ and WSH) with the Newcastle-Ottawa quality assessment scale (NOS) range from 0 to 8 which is used in the evaluation of non-randomized studies. Studies with an NOS score ≥6 were assigned as high-quality studies. Studies from conference abstracts and with score of zero were defined as low-quality studies. Discordant studies were evaluated by the two reviewers together.

### Statistical analysis

The main outcome measures for this meta-analysis were rates of progression. The primary aim was to search the effect of p53 status (positive or negative) detected by IHC on progression rates. For dichotomous data, a summary risk ratio (RR) and its 95 % CI were calculated. An observed HR >1 implied progression for the study group with positive p53, relative to the negative group. Subgroup analyses were performed to examine if our pooled estimate of the prognostic value was influenced by a test of heterogeneity of the combined HRs, carried out using the Chi-square test and *I*-squared statistic. A *P* > 0.10 for the Chi-square test and an *I*^2^ value of <25 % was considered to represent a low level of heterogeneity between studies. *I*^2^ > 50 % indicated large heterogeneity among studies, whereas *I*^2^ values between 25 and 50 % indicated moderate heterogeneity [16]. Sensitivity analysis was also performed by removing one study at a time to calculate the overall homogeneity and effect size; the Galbraith plot was also performed to examine the possible distinct articles. Publication bias was estimated with a visual inspection of funnel plots. All 95 % CIs were two-sided. The meta-analysis was performed using Review Manager (RevMan) software version 5.2 (RevMan 5.2; the Nordic Cochrane Center, the Cochrane Collaboration, Copenhagen, Denmark).

## Results

### Study eligibility

**Fig. 1:**
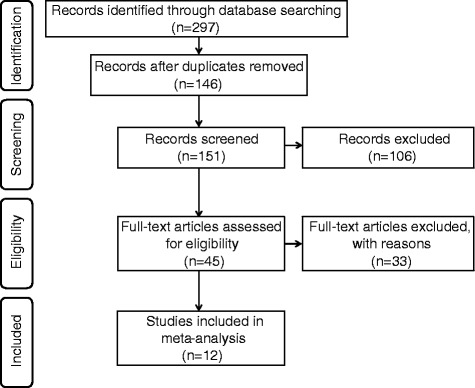
Flowchart of study selection

Through electronic screening, 151 potentially relevant articles consistent with our searching terms were identified and 106 of them were eliminated after reading the abstracts. Of the 45 article that were in full-text format, 24 were excluded because of not including progression data; 9 were excluded since there were no specific result about p53 status in T1 NMIBC. The flowchart of selecting the procedure and the exclusive reason of studies is summarized in Fig. 1. A total of 12 studies were included in the meta-analysis (Table 1).

**Table 1:** Studies included in the meta-analysis

Study	Year	Country	p53 (+)	p53 (−)	No. of patients	Cutoff (%)	Antibody	Intravesical therapy	Quality score
Park [21]	2013	Korea	32	29	61	10	DO7	Pure BCG	7
Dalbagni [14]	2007	USA	53	36	89	20	Pab1801	Non-pure	5.5
Saint [12]	2004	France	24	78	102	20	DO7	Pure BCG	6
Lopez-Beltran [29]	2004	Spain	22	29	51	6	Pab1801	Pure BCG	7
Gil [15]	2003	Spain	40	27	67	20	DO7	Non-pure	7
Peyromaure [22]	2002	France	18	11	29	20	DO7	Pure BCG	6
Liopis [13]	2000	Spain	24	56	80	20	Bp-53-12-1	Non-pure	8
Shariat [30]	2000	USA	26	10	36	10	DO7	Non-pure	7
Toktas [31]	1999	Turkey	18	34	36	10	DO7	Non-pure	6
Pages [23]	1998	France	27	16	43	10	DO7	Pure BCG	6
Vatne [32]	1995	Norway	40	19	59	20	DO1	Non-pure	7
Sarkis [8]	1993	USA	25	18	43	20	Pab1801	Non-pure	7

### Quality assessment

Quality assessment of the 12 studies included in the meta-analysis was performed by using NOS. In this quality assessment system, scores 0–3, 4–5, and 6–8 are accepted as low, medium, and high quality, respectively. The median score of the studies included in the meta-analysis was found as 7.

### Heterogeneity assessment

Meta-analysis of the correlation between p53 overexpression with progression of T1 NMIBC resulted in *P* < 0.0001 and *I*^2^ = 39 %, suggesting that there was moderate heterogeneity and that the subgroup analysis should be carried out. Hence, we carried out subgroup analysis according to stage, intravesical therapy, antibody clone, cutoff value of positivity of p53, and ethnicity. However, none of these factors was found to be significantly correlated to heterogeneity using subgroup analysis among the studies.

### Meta-analysis results

#### Pooled analysis

##### p53 overexpression and progression of T1 NMIBC

**Fig. 2:**
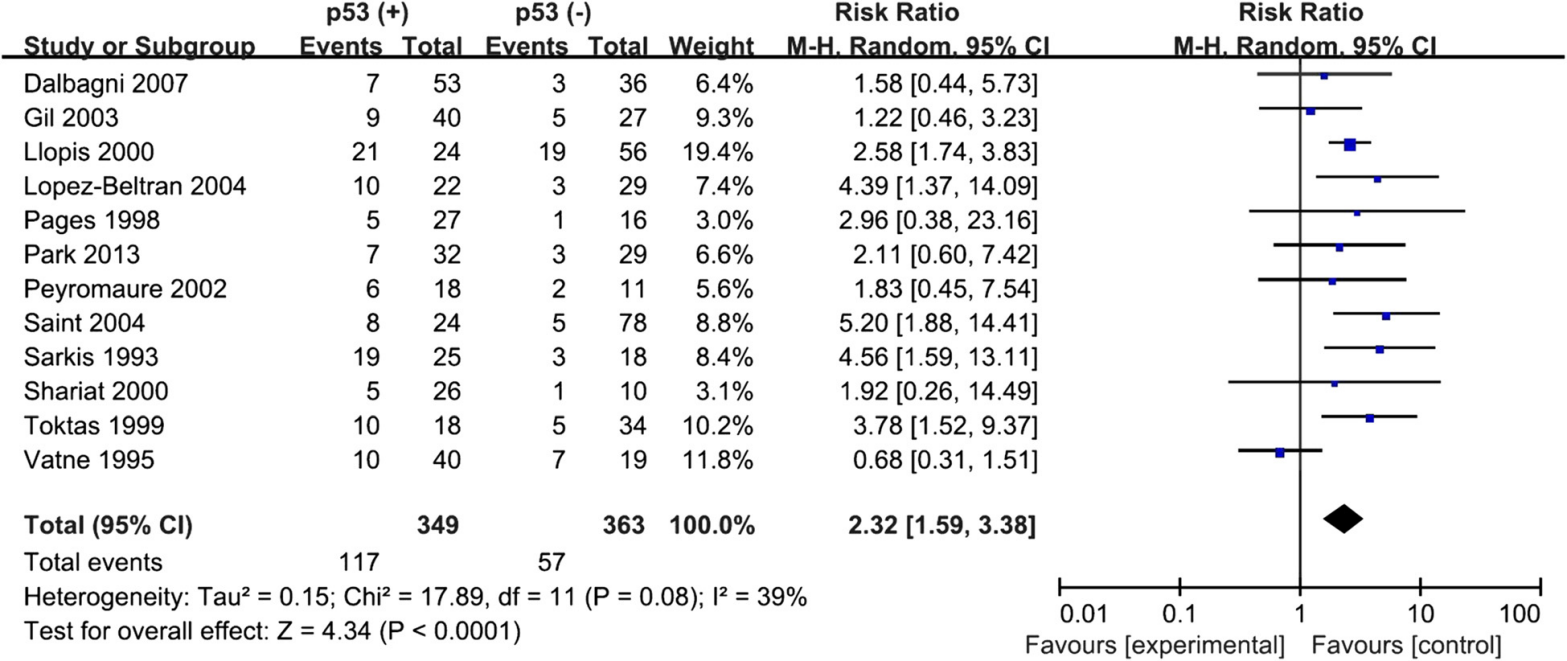
Forest plots of RRs estimated for the relationship between p53 overexpression and progression of T1 NMIBC patients

There were 12 studies with a total of 712 patients included in final analysis to assess the effect of p53 overexpression on progression of T1 NMIBC. As shown in Fig. 2, the pooled RR was 2.32 (95 % CI 1.59–3.38) by random-effects model for the existence of a moderate heterogeneity (*I*^2^ = 39 %, *P* < 0.0001), which suggested p53 overexpression was associated with progression of T1 NMIBC. Subgroup analysis according to grade, intravesical therapy, antibody clone, cutoff value of positivity of p53, and ethnicity was also performed (Table 2). The results suggested that all of the relevant stratified factors did not have a significant correlation with heterogeneity (*P* ranging from 0.18 to 0.63) and did not alter the significant prognostic impact of p53 overexpression.

**Table 2:** Summarized RRs of overall and subgroup analysis for T1 NMIBC patients with p53 overexpression

Variables	No. of studies	Random effects RR (95 % CI)	Heterogeneity		Subgroup analysis *P* value
			*I* ^2^ (%)	*P* value	
Overall	12	2.41 (1.55, 3.75)	42	0.06	<0.0001
Ethnicity					0.44
Asian	2	3.09 (1.48, 6.46)	0	0.003	
Non-Asian	10	2.20 (1.41, 3.45)	46	0.0005	
Stage					0.63
T1G3	3	2.71 (1.31, 5.64)	0	0.007	
All NMIBC	9	2.24 (1.40, 3.59)	52	0.0008	
Intravesical therapy					0.18
Pure BCG	5	3.35 (1.89, 5.93)	0	<0.0001	
Non-pure	7	1.97 (1.17, 3.33)	57	0.03	
Antibody					0.49
DO7	7	2.59 (1.64, 4.08)	0	<0.0001	
Pab1801	3	3.38 (1.73, 6.60)	0	0.0003	
Others	2	1.38 (0.36, 5.34)	89	0.002	
Cutoff					0.41
20 %	7	2.68 (1.92, 3.74)	9	<0.0001	
Others	5	1.83 (0.78, 4.26)	51	0.16	

#### Subgroup analysis

##### p53 overexpression and progression of T1G3 NMIBC

There were 3 studies that included a total of 141 pure T1 grade 3 (T1G3) NMIBC patients; the RR was 2.71 (95 % CI 1.31–5.64) by random-effects model for the existence of no heterogeneity (*I*^2^ = 0 %, *P* = 0.007), which suggested p53 overexpression was associated with progression of T1G3 NMIBC (Table 2).

##### p53 overexpression and progression of T1 NMIBC treated with BCG

There were 4 studies that included a total of 286 pure T1 NMIBC patients treated with Bacillus Calmette-Guérin (BCG) intravesical therapy; the RR was 2.71 (95 % CI 1.31–5.64) by random-effects model for the existence of no heterogeneity (*I*^2^ = 0 %, *P* < 0.0001), which suggested p53 overexpression was associated with progression of T1 NMIBC patients treated with BCG intravesical therapy (Table 2).

### Publication bias assessment and sensitivity analysis

**Fig. 3:**
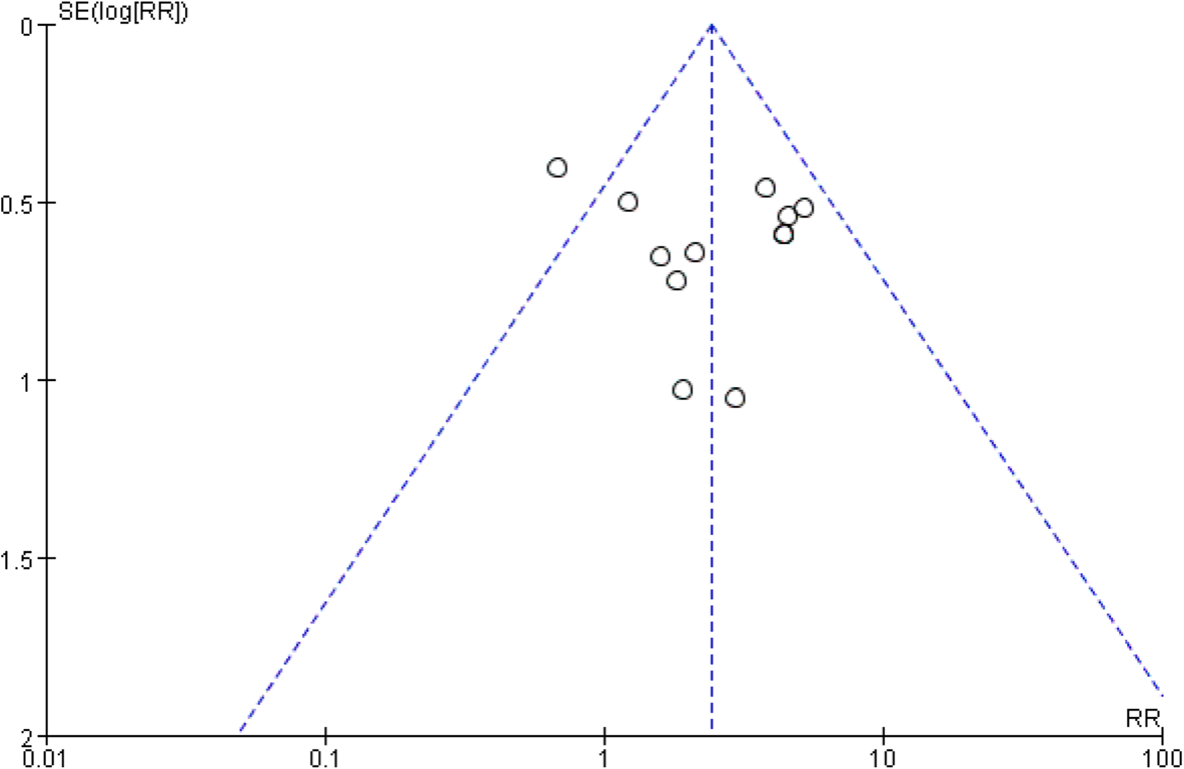
Publication bias determination using funnel plot

Investigation of bias by a funnel plot showed no evidence of significant publication bias among the studies with respect to the effect of p53 status on progression of T1 NMIBC (Fig. 3).

**Fig. 4:**
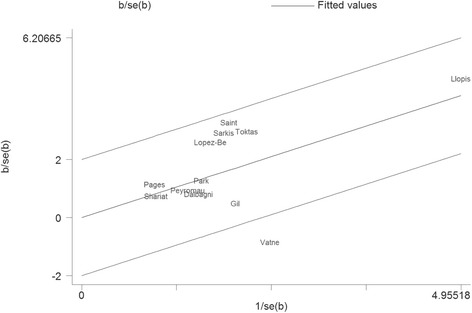
Galbraith plot of association between p53 overexpression and T1 NMIBC patient progression risk

Sensitivity analysis was conducted to assess the influence of individual study on the pooled effect. One study by Vatne et al. was identified in the Galbraith plot as the outliers (Fig. 4). When we removed the study by Vatne et al., the initial heterogeneity (*I*^2^ = 39 %, *P* < 0.0001) was reduced to none (*I*^2^ = 0 %, *P* = 0.63) in evaluating the association of p53 overexpression and progression of T1 NMIBC patients (Table 3).

**Table 3:** Results of sensitivity analysis

Sensitivity analysis	Heterogeneity		Pooled analysis	
	*I* ^2^ (%)	*P* value	RR (95 % CI)	*P* value
Include Vatne et al.’s study	39	0.08	2.32 (1.59, 3.38)	<0.0001
Exclude Vatne et al.’s study	0	0.67	2.74 (2.05, 3.65)	<0.0001

## Discussion

T1 NMIBC are potentially more lethal because approximately 40 % of tumors managed conservatively progress to MIBC or develop distant metastasis within 5 years [3]. Molecular markers are promising for accurately predicting the progression of T1 NMIBC patients. p53 plays an important role in the regulation of cell cycle and apoptosis. To date, the majority of available clinical reports have involved small sample sizes and given conflicting results, therefore unable to determine the value of p53 status in predicting progression of T1 NMIBC. Thus, we conducted a meta-analysis of 12 studies to systematically evaluate the association between p53 status and progression of T1 NMIBC.

To the best of our knowledge, this is the first meta-analysis to evaluate the relationship between p53 status and the progression of T1 NMIBC. In the overall pooled analysis of the association of p53 status with progression of T1 NMIBC, the results suggested that p53 overexpression associated with progression of T1 NMIBC.

In the test of heterogeneity, there was a moderate heterogeneity (*I*^2^ = 39 %) in the analysis of the association of p53 status with progression of T1 NMIBC. Although the data were aggregated using the random-effects models, heterogeneity among the studies continued to be a potential problem to influence the reliability of pooled results. Hence, we carried out subgroup analysis according to grade, intravesical therapy, antibody clone, cutoff value of positivity of p53, and ethnicity. However, none of these factors was found to be significantly correlated to heterogeneity using a subgroup analysis.

However, there is no heterogeneity in T1G3 NMIBC (*I*^2^ = 0 %); it suggested that p53 overexpression was associated with incremental progression of T1G3 NMIBC. T1G3 NMIBC is one subtype of NMIBC, with the highest progression risk [17]. Because T1G3 NMIBC can present clinical characteristics of invasive tumors and clinical T1 tumors contain a variable proportion of understaged pT2 tumors, long-term rates of cancer-specific mortality reach up to 34 % [18]. Expert recommendations on the optimal treatment strategy for patients with T1G3 NMIBC range from conservative intravesical therapy to early radical cystectomy [18, 19]. Underestimation of the potential for progression in T1G3 NMIBC could lead to unpreventable morbidity and mortality; however, patients treated with early radical cystectomy faced increased perioperative mortality and decreased quality-of-life. Therefore, T1G3 NMIBC patients with p53 overexpression may probably obtain better benefit from early radical cystectomy.

In addition, we did not observe heterogeneity in patients treated with BCG intravesical therapy (*I*^2^ = 0 %); it suggested that p53 overexpression was associated with elevated progression of T1 NMIBC received by BCG intravesical therapy. Several investigators have evaluated the prognostic value of nuclear p53 immunoreactivity before BCG intravesical therapy. A correlation between pretreatment p53 overexpression and disease progression after BCG therapy was found in several studies [12, 20]. Other authors found no such correlation [21–26]. Nevertheless, it was found that BCG treatment was less likely to be successful in patients with mutated p53 by using yeast functional assay [27]. To date, whether p53 tumor status is an independent predictive factor of BCG response in T1 NMIBC still remains a debate. The present meta-analysis suggested that T1 NMIBC patients with p53 overexpression have increased progression risk after BCG intravesical therapy; early radical cystectomy could be considered in these patients.

It should be noted that our meta-analysis has several limitations. First, this meta-analysis was limited by the presence of heterogeneity across the studies. The heterogeneity possibly caused by the differences in the characteristics of the patients, IHC technique, cutoff values, and follow-up time. Second, to reduce the bias from different detection methods, we only included the studies measuring p53 expression by IHC. IHC has been widely used to detect molecular markers, because the method is simple, fast, and reliable [28]. However, the differences of the clones of antibody, concentration, and cutoff value used in different studies might also cause potential bias. Especially, the cutoff used to define p53 overexpression is probably of prime importance. In the present meta-analysis, most of the studies chose a cutoff value of 20 %; p53 overexpression was a predictor of progression in T1 NMIBC, and there is low heterogeneity among these studies (*I*^2^ = 9 %, *P* < 0.0001). It suggested that a cutoff value of 20 % may be a proper cutoff of p53. Furthermore, the interpretation of immunohistochemical results may vary among different centers. It is, therefore, necessary to define a standard evaluation system to promote the application of p53 detected by IHC in clinical practice. Third, the meta-analysis may have been influenced by publication bias, as we limited the literature search to studies performed in English. Finally, most of the included studies were observational trials, which contained more potential confounders and provided the lower level of evidence compared with randomized controlled studies. Therefore, the results should be interpreted cautiously.

## Conclusions

The present study is the first meta-analysis to quantitatively assess the association between p53 status and progression of T1 NMIBC. The results showed that p53 overexpression might be a useful predictive biomarker for evaluating progression in T1 NMIBC patients, especially in T1G3 NMIBC and patients treated by BCG intravesical therapy. However, to strengthen our findings, further larger prospective studies with better standardized methods are needed to make a comprehensive conclusion on the prognostic role of p53 overexpression in T1 NMIBC.

## Ethical approval

This article does not contain any studies with human participants performed by any of the authors.

## Abbreviations

NMIBC: non-muscle invasive bladder cancer
T1G3: T1 grade 3
